# The Nuclear Receptor Genes *HR3* and *E75* Are Required for the Circadian Rhythm in a Primitive Insect

**DOI:** 10.1371/journal.pone.0114899

**Published:** 2014-12-11

**Authors:** Yuichi Kamae, Outa Uryu, Taiki Miki, Kenji Tomioka

**Affiliations:** 1 Graduate School of Natural Science and Technology, Okayama University, Okayama, 700–8530, Japan; 2 Department of Biology, Faculty of Science, Okayama University, Okayama, 700–8530, Japan; University of Würzburg, Germany

## Abstract

Insect circadian rhythms are generated by a circadian clock consisting of transcriptional/translational feedback loops, in which CYCLE and CLOCK are the key elements in activating the transcription of various clock genes such as *timeless* (*tim*) and *period* (*per*). Although the transcriptional regulation of *Clock* (*Clk*) has been profoundly studied, little is known about the regulation of *cycle* (*cyc*). Here, we identify the orphan nuclear receptor genes *HR3* and *E75*, which are orthologs of mammalian clock genes, *Rorα* and *Rev-erbα*, respectively, as factors involved in the rhythmic expression of the *cyc* gene in a primitive insect, the firebrat *Thermobia domestica*. Our results show that *HR3* and *E75* are rhythmically expressed, and their normal, rhythmic expression is required for the persistence of locomotor rhythms. Their RNAi considerably altered the rhythmic transcription of not only *cyc* but also *tim*. Surprisingly, the RNAi of *HR3* revealed the rhythmic expression of *Clk*, suggesting that this ancestral insect species possesses the mechanisms for rhythmic expression of both *cyc* and *Clk* genes. When either *HR3* or *E75* was knocked down, *tim*, *cyc*, and *Clk* or *tim* and *cyc*, respectively, oscillated in phase, suggesting that the two genes play an important role in the regulation of the phase relationship among the clock genes. Interestingly, *HR3* and *E75* were also found to be involved in the regulation of ecdysis, suggesting that they interconnect the circadian clock and developmental processes.

## Introduction

Circadian clocks provide an adaptive advantage by coordinating physiological, behavioral, and biochemical events to occur at an appropriate time of the day [1]. The clock is believed to consist of transcriptional/translational feedback loops. In the fruit fly *Drosophila melanogaster*, molecular biological studies have shown that the transcriptional activators CLOCK (CLK) and CYCLE (CYC) heterodimerize and bind to the E-box within the promoters of the *per* and *tim* genes to activate their transcription [2]–[4]. PER and TIM proteins accumulate and, subsequently, associate with the CLK/CYC complex to repress their own transcription, forming an about 24-h period feedback loop for the rhythmic expression of *per* and *tim*. In addition, the transcription of *Clk* is also under circadian regulation [5], [6]. It is controlled by cyclical and reciprocal activities of the basic leucine zipper transcription factors VRILLE (VRI) and PAR DOMAIN PROTEIN 1ε (PDP1ε). Both VRI and PDP1ε competitively bind to the VRI/PDP1ε binding site (V/P-box) within the *Clk* promoter to repress or activate transcription, respectively [7]. Mutations in *vri* and *Pdp1ε* affect locomotor activity rhythms, as well as the CLK expression levels [6], [8]. The expression of *vri* and *Pdp1ε* is under circadian control, which requires CLK/CYC, thereby connecting the PER/TIM and VRI/PDP1ε feedback loops.

The firebrat *Thermobia domestica* also possesses a circadian clock based on the rhythmic expression of *tim* under the control of *Clk* and *cyc* [9], [10]. However, unlike *Drosophila*, *cyc*, but not *Clk*, was rhythmically expressed in both light–dark cycles (LD) and constant darkness (DD) [9]. Similar patterns of *cyc* and *Clk* expression have been reported for *Gryllus bimaculatus* and *Apis mellifera* [11]–[13]. While a large body of genetic and biochemical evidence has been accumulated on the regulation of *per*, *tim*, and *Clk* expressions, little is known about the control of *cyc*. In the mammalian clock, the *cyc* ortholog *Bmal1* is cyclically expressed, and the underlying mechanism has been extensively studied. Two nuclear receptors, RORα and REV-ERBα, are known to be involved in the mechanism. They directly regulate the expression of *Bmal1* by binding to a specific ROR/REV-ERB response element in the *Bmal1* promoter region [14]. RORα stimulates *Bmal1* transcription, whereas REV-ERBα represses it [15], [16]. Therefore, we expected that *cyc* might be regulated by a mechanism similar to that found in mammals.

In this study, by taking advantage of the efficacy of RNAi, we investigated the role of probable *nuclear hormone receptor 3* gene (*HR3*), a *Ror* ortholog, and *ecdysone induced protein 75* gene (*E75*), a *Rev-erb* ortholog, for the first time in the insect circadian clock by using the firebrat, one of the most primitive insect species. Surprisingly, the gene silencing of *HR3* by RNAi revealed the rhythmic expression of *Clk*, suggesting that this ancestral insect possesses a mechanism for the rhythmic expression of both *cyc* and *Clk* genes. The RNAi of *HR3* and *E75* disrupted the expression levels and phase relationship of the clock genes, leading to a loss of the locomotor activity rhythm. *HR3* and *E75* were also found to be involved in the regulation of ecdysis. Based on the obtained results, we propose a unique clock model for this ancestral insect.

## Materials and Methods

### Animals

Adult firebrats, *Thermobia domestica*, were used for all experiments. They were taken from our laboratory culture reared under a light cycle of 12-h light and 12-h dark conditions (LD 12∶12) at a constant temperature of 30°C. They were fed laboratory chow (CA-1, Clea Japan, Tokyo).

### cDNA cloning of *HR3* and *E75*

Total RNA was extracted with the TRIzol Reagent (Invitrogen, Carlsbad, CA) from 5 adult firebrats collected at zeitgeber time (ZT) 10 (ZT0 corresponds to light on and ZT12 to light off). A 5 µg of total RNA was used for reverse transcription to obtain cDNA, using SuperScript III (Invitrogen). Using the obtained cDNA as a template, we performed PCR with degenerate primers deduced from the conserved amino acid sequences among insect *HR3* and *E75* homologs (Table 1). The amplified fragments were cloned into T-Vector pMD20 (Takara, Ohtsu, Japan) and sequenced with the BigDye Terminator v.3.1 Cycle Sequencing Kit (Applied Biosystems, Foster City, CA). The 5′ and 3′ RACEs were performed with the GeneRacer Kit (Invitrogen) and SMARTer RACE cDNA Amplification Kit (Takara), respectively, using Blend Taq Plus (Toyobo, Osaka, Japan) with gene-specific primers as follows: 5′-TTCCTCGGGCACTGATAGTT-3′ for 5′RACE, and 5′-CGAGGTTCGGTTTCACAGAG-3′ for 3′RACE of *HR3*, and 5′-GACGCCTGCTTTGAGAAGAG-3′ for 5′RACE, and 5′-AAGCTGGACTCGCCTAATGA-3′ for 3′RACE of *E75*. RACE fragments were purified, cloned, and sequenced as mentioned above. Sequences were analyzed by Genetyx v.6 (Genetic Information Processing Software, Tokyo, Japan) and BioEdit v.7.0.9.0 (Biological Sequence Alignment Editor, Ibis Therapeutic, Carlsbad, CA). Amino acid sequences of *HR3* and *E75* were analyzed, and a neighbor-joining tree was inferred with ClustalW (http://clustalw.ddbj.nig.ac.jp/). Known sequences of insects were obtained from GenBank.

**Table 1:** PCR primers used for PCR, quantitative RT-PCR, and dsRNA synthesis.

Genes	Forward	Reverse
Degenerate PCR		
*HR3*	5′-ccagtcctccgtggtgaaYtaYcaRtg-3′	5′-cggggaccatcttggcRaaYtcDat-3′
*E75*	5′-cgcatcctggccgcNatgcaRca-3′	5′-acttcttgtggggcttgtaggYYtcRtccat-3′
Quantitative RT-PCR		
*HR3*	5′-CGAGGTTCGGTTTCACAGAG-3′	5′-GAGGAGGACGGTTGTTGTTG-3′
*E75*	5′-GAAATGCCCAGCTACACCAC-3′	5′-GAACTCAACAACCCCACGAA-3′
*Timeless*	5′-TACAAGCCAGGTCCATCACA-3′	5′-TCAAGCGTCAATTCAGCATC-3′
*Clock*	5′-ATCGCAAGGGTCTGGAAGTG-3′	5′-GGAAAACTCGCCAAGACAGG-3′
*Cycle*	5′-CGTGTAATCTGTCGTGTTTGGTG-3′	5′-GAATCGTCCGCCTTTCCTC-3′
*rp49*	5′-AGTCCGAAGGCGGTTTAAGG-3′	5′-TACAGCGTGTGCGATCTCTG-3′
dsRNA synthesis		
*HR3#1*	5′-TAATACGACTCACTATAGGGACTTCTCCTGCCTCGGGTAT-3′	5′-TAATACGACTCACTATAGGGCAGTTTTAGCTCCGCCAAAC-3′
*HR3#2*	5′-TAATACGACTCACTATAGGGCGAAAATGGTACCTGGCTTT-3′	5′-TAATACGACTCACTATAGGGTCGCTTGAATTTACCCAAGG-3′
*E75#1*	5′-TAATACGACTCACTATAGGGAGTCAAAGTCACCCCGAGAA-3′	5′-TAATACGACTCACTATAGGGTCATTAGGCGAGTCCAGCTT-3′
*E75#2*	5′-TAATACGACTCACTATAGGGCAGAACCAATGGTGGTTTCC-3′	5′-TAATACGACTCACTATAGGGGCCGCTGTTGTTGTAGAGGT-3′
*timeless*	5′-TAATACGACTCACTATAGGGTGCATTTGGTTGTGACTGCT-3′	5′-TAATACGACTCACTATAGGGAGAGGCGTGTGCCTTGTACT-3′
*Clock*	5′-TAATACGACTCACTATAGGGACCACCAATCGAAAAATGGA-3′	5′-TAATACGACTCACTATAGGGCCCAGTTCCCACGAAAACTA-3′
*cycle*	5′-TAATACGACTCACTATAGGGAGGGGCTGTTCATTCCTACA-3′	5′-TAATACGACTCACTATAGGGCGCCCACGACTTCAAATAAC-3′
*DsRed2*	5′-TAATACGACTCACTATAGGGTCATCACCGAGTTCATGCG-3′	5′-TAATACGACTCACTATAGGGCTACAGGAACAGGTGGTGGC-3′

### Quantitative real-time RT-PCR

Quantitative reverse-transcription PCR (qPCR) was used to measure mRNA levels. Total RNA extraction from adult firebrats was performed using the TRIzol Reagent, and the obtained RNA was treated with DNase I to remove contaminating DNA. Approximately 500 ng of total RNA of each sample was reverse transcribed with random 6mers using the PrimeScript RT Reagent Kit (Takara). Real-time PCR was performed with the Mx3000P Real-Time PCR System (Stratagene, La Jolla, CA) using the Universal SYBR Green Master (Roche, Tokyo, Japan), including SYBR Green with primers for *HR3*, *E75*, *tim* (GenBank/EMBL/DDBJ Accession No. AB644410), *Clk* (AB550828), *cyc* (AB550829), and *rp49* (AB550830) (Table 1). In all cases, a single expected amplicon was confirmed by melting analysis. The results were analyzed using the software associated with the instrument. The quantification of mRNA levels was performed by the standard curve method, and the values were normalized with those for *rp49*, a housekeeping gene, at each time point. Results of 3–4 independent experiments were pooled to calculate the mean ± SEM. Data were analyzed by *t*-test or ANOVA.

### RNA interference

Double-stranded RNAs (dsRNA) for *HR3*, *E75*, *tim*, *Clk*, and *cyc* were synthesized using the MEGAscript High Yield Transcription Kit (Ambion, Austin, TX) as described previously [10]. *DsRed2* derived from a coral species (*Discosoma* sp.) was used for negative control because the firebrat does not possess the gene; its dsRNA was synthesized in the same procedure. Primers fused with the T7 promoter sequence were designed for synthesizing the dsRNAs (Table 1). The dsRNA solution was stored at −80°C until use. A total of 69 nl (10 µM) of the dsRNA solution was injected using the Nanoliter Injector (WPI, Sarasota, FL) into the abdomen of adult firebrats anesthetized with CO_2_. The injected amount of dsRNA solution corresponded to 10.23–13.95 µg/g (body weight). The total RNA was extracted from the whole body 1 week after the injection when the effect of the RNA was expected to reach its maximal potential [17].

### Recording of locomotor activity

To monitor locomotor activity, adult firebrats were individually housed in transparent acrylic rectangular tubes (6×6×70 mm) as previously described [10]. The raw data were displayed as conventional double-plotted actograms to judge the activity patterns and existence of the rhythmicity, and free-running periods were analyzed by the Lomb-Scargle periodogram by using actogramJ [18], [19]. If the peak of the periodogram appeared above the 0.05 confidence level, then the period of the peak was designated as statistically significant.

### Hormonal treatment

20 hydroxyecdysone (20E) (Sigma, St Louis, MO, USA) was dissolved in 100% EtOH (0.5 mg/ml) and 69 nl (34.5 ng) was injected into the abdomen of adult firebrats anesthetized with CO_2_. The control was treated with an equivalent amount of 100% EtOH. The total RNA from whole body was isolated at 1week after the injection to measure the mRNA levels of *HR3* and *E75*.

## Results

### Cloning and sequencing of *HR3* and *E75* cDNA

**Figure 1:**
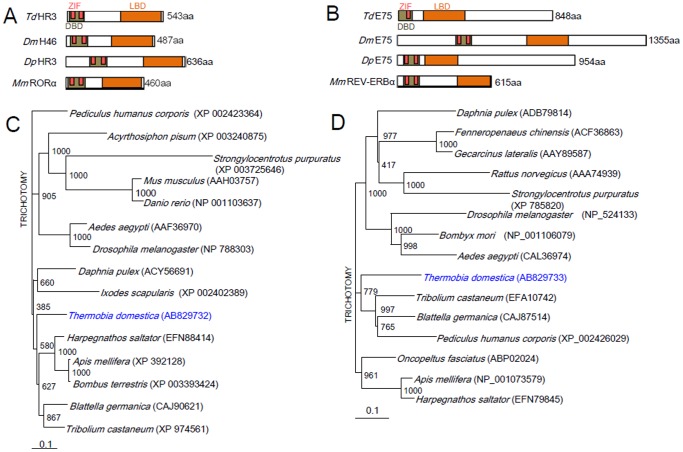
Sequence alignments of conserved domains and phylogenetic neighbor-joining tree of *HR3* and *E75*. (A, B) Schematic structure of various HR3 (A) or E75 (B) proteins, comparing the organization of the 3 conserved domains, DNA-binding domain (DBD), zinc finger domain (ZIF), and ligand binding domain (LBD). The numbers on the right indicate the number of amino acid residues. *Td, Thermobia domestica*; *Dm, Drosophila melanogaster*; *Dp, Danaus plexippus*; *Mm, Mus musculus*. (C, D) A phylogenetic neighbor-joining tree of known animal HR3 or E75 proteins. The GenBank or RefSeq accession numbers are indicated in brackets. A reference bar indicates distance as the number of amino acid substitutions per site.

The cDNAs of the nuclear receptors *HR3* and *E75* were obtained from *T. domestica* (GenBank Accession Nos. AB829732 for *HR3* and AB829733 for *E75*). The *T. domestica HR3* (*Td'HR3*) and *Td'E75* cDNAs encode proteins of 543 and 848 aa residues, respectively, and have 5′- and 3′-untranslated regions (UTR) of 61 and 228 bp, and 98 and 102 bp, respectively. Fig. 1 shows the alignment of the obtained amino acid sequences of *Td'*HR3 and *Td'*E75 with those of several other species. *Td'*HR3 shares 74% sequence identity with *Tribolium castaneum* HR3 (*Tc'*HR3). Its DNA-binding domain (DBD) contains two zinc fingers that share 95–100% identity with the HR3 orthologs from other species (Table 2). The ligand-binding domain (LBD) shared 61–83% identity with those of other insects' HR3 orthologs. *Td'*E75 shared 45–61% identity with other insects' E75 orthologs and its DBD and LBD were nearly 100% and 69–88% identical, respectively, to those of the E75 orthologs. The DBD was equipped with a zinc finger motif, which shares 100% identity (Fig. 1B; Table 2). These are the first *HR3* and *E75* orthologs in Thysanura insects.

**Table 2:** Overall amino acid identity and similarity (%) of whole sequence and functional domains of *Td'*HR3 and *Td'*E75 with their insect homologs.

Species	Identity	Similarity	Identity			
			DBD	Zinc Finger 1	Zinc Finger 2	LBD
**HR3**						
*Daphnia pulex*	65	73	97	100	95	78
*Drosophila melanogaster*	61	76	97	100	95	61
*Pediculus humanus corporis*	66	74	100	100	100	83
*Tribolium castaneum*	74	84	100	100	100	83
**E75**						
*Daphnia pulex*	55	68	100	-	100	77
*Drosophila melanogaster*	49	61	100	-	100	69
*Pediculus humanus corporis*	55	65	100	-	-	87
*Tribolium castaneum*	61	68	100	-	100	88

DBD, DNA-binding domain; LBD, ligand-binding domain.

A phylogenetic tree based on the amino acid sequences of *HR3* and *E75* from known insects, sea urchins, and some vertebrates revealed three clusters of *HR3* and *E75*. *Td'HR3* formed a cluster with the beetle *Tribolium castaneum*, the cockroach *Blattella germanica*, the honeybee *Apis mellifera*, the bumble bee *Bombus terrestris*, and the ant *Harpegnathos saltator*, while *Td'E75* with the beetle *Tribolium castaneum*, the cockroach *Blattella germanica*, and the louse *Pediculus humanus corporis* (Fig. 1).

### Temporal expression patterns of *Td'HR3* and *Td'E75* mRNA

**Figure 2:**
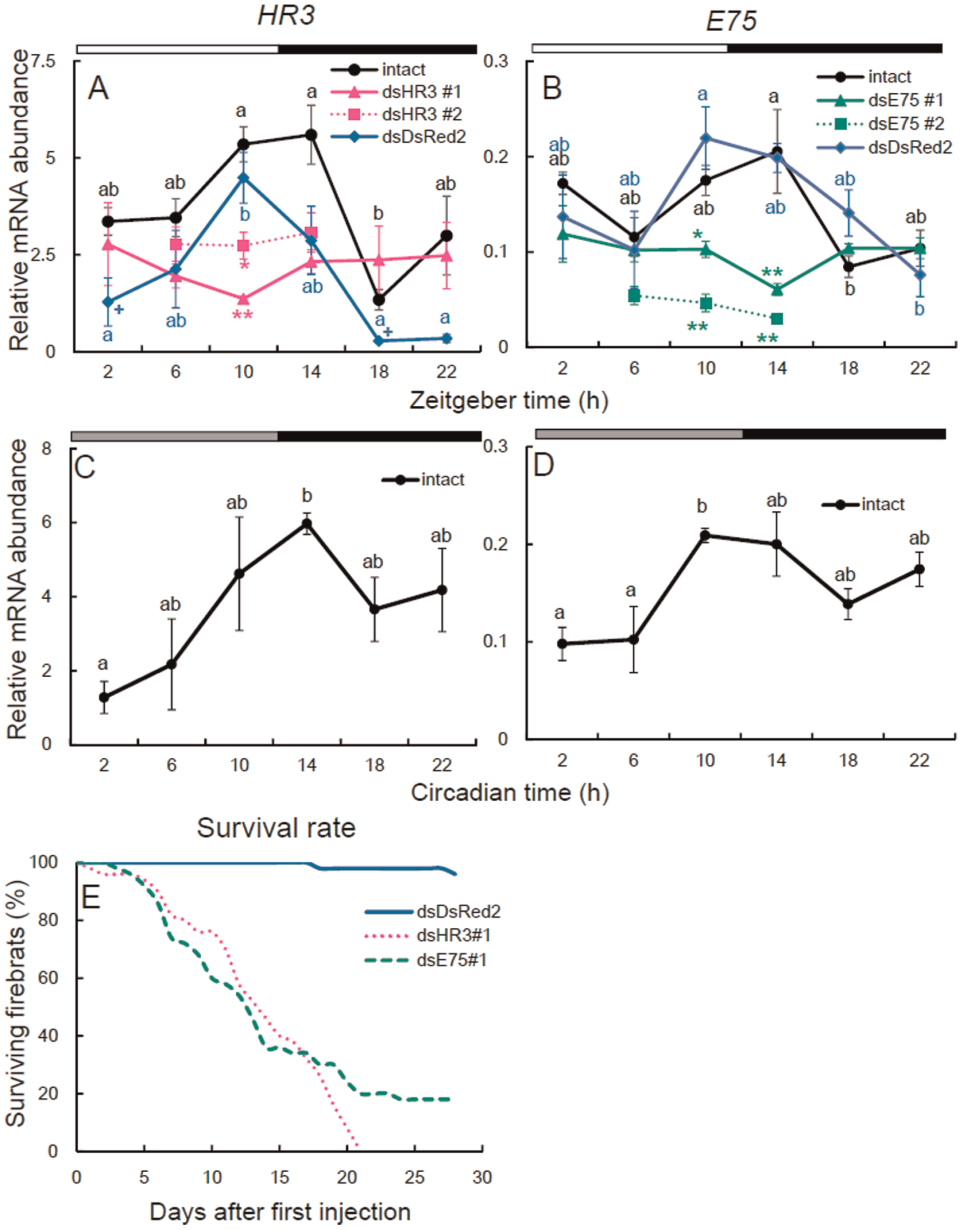
Expression profiles of *Td'HR3* and *Td'E75* mRNA and effects of their dsRNAs on their mRNA levels and the survival rate in firebrats. Both *Td'HR3* (A, C) and *Td'E75* (B, D) were rhythmically expressed in LD (A, B) and DD (C, D), peaking late (subjective) day to early (subjective) night (black symbols). Ds*HR3#1,2* (pink triangles and squares for #1 and #2, respectively) and ds*E75#1,2* (green triangles and squares for #1 and #2, respectively) downregulated mRNA levels and disrupted the rhythm of their respective genes (A and B). Treatment with ds*DsRed2* as a negative control did not disrupt the rhythmic expression in both genes (A and B, blue symbols), but caused a significant decrease in *Td'HR3* mRNA levels at ZT 2 and ZT 18 (+ *P*<0.05, *t* test vs intact). White, black, and gray bars above each graph indicate day, night/subjective night, and subjective day, respectively. Total RNA was extracted from firebrats collected at 4-h intervals starting 2 h after lights on or 2 h into subjective day (ZT2 or CT2, respectively). The data collected from 3–4 independent experiments were averaged and plotted as the mean ± SEM values relative to the value of *rp49* mRNA used as the reference. Values with different letters significantly differ from each other within the same treatment groups (*P*<0.05, ANOVA with Tukey test, S1 Table). *P<0.05, **P<0.01, *t* test vs ds*DsRed2* (exact *P* values are shown in S2 Table). (E) Survival rate after injection of 10.23–13.95 µg/g ds*HR3*, ds*E75*, or ds*DsRed2* into the abdomen. Ds*HR3* and ds*E75* significantly reduced the survival rate. For further explanation, see text.

To determine whether the transcripts of *Td'HR3* and *Td'E75* oscillated in a circadian manner, we measured the levels of their mRNA under LD 12∶12 and on the 2^nd^ day of DD by using qPCR (Fig. 2). In LD, both *Td'HR3* and *Td'E75* mRNAs were rhythmically expressed, peaking at late day or early night (ANOVA, *P*<0.03, S1 Table). Their rhythmic expressions persisted in DD (ANOVA, *P*<0.05, S1 Table), thus, suggesting their circadian control.

### RNAi of *Td'HR3* and *Td'E75*

To examine the possible role of *Td'HR3* and *Td'E75* in the firebrat circadian clock, we examined the effectiveness of RNAi on *Td'HR3* and *Td'E75* one week after the treatment. We first examined the effects of dsRNA of *DsRed2* used as negative control. As shown in Fig. 2A, *Td'HR3* was slightly reduced but the reduction was not significant at most time points, except for ZT 2 and ZT 18. No significant effect was observed in *Td'E75* transcript levels (Fig. 2B).

For RNAi of *Td'HR3* and *Td'E75*, we used two different dsRNAs synthesized for different regions of the two genes (Table 1). The *Td'HR3* RNAi and *Td'E75* RNAi firebrats showed a high lethality because of ecdysis failure (Fig. 2E); 50% of the treated firebrats died within two weeks. This may be because these genes are involved in the ecdysone-signaling pathway involved in ecdysis. When examined 1 week after dsRNA treatment, the dsRNAs significantly reduced respective mRNAs (Fig. 2A–B, S2 Table) to near or below basal levels of intact firebrats and the levels were maintained throughout the day with no rhythmic changes (ANOVA, *P*>0.1, S1 Table). Thus, the expression of *Td'HR3* and *Td'E75* were successfully suppressed through RNAi. The levels of knockdown were similar to those known for *Td'Clk* and *Td'cyc* but less than for *Td'tim* [9], [10].

### *Td'HR3* dsRNA and *Td'E75* dsRNA treatments disrupt circadian locomotor activity and ecdysis

**Figure 3:**
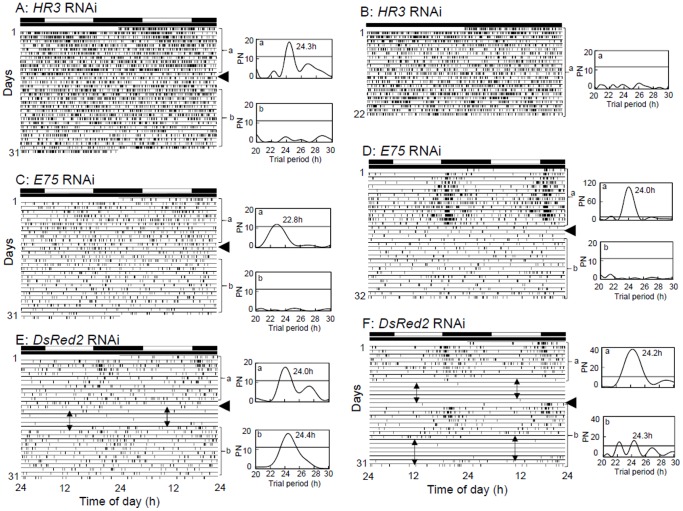
Effects of *HR3* and *E75* dsRNA on locomotor rhythms in the firebrat. Double-plotted actograms (left) and Lomb-Scargle periodograms (right) of locomotor rhythms under LD 12∶12 and DD at a constant temperature of 30°C are shown for firebrats injected with *HR3* (A, B) and *E75* dsRNA (C, D) or *DsRed2* dsRNA (E, F). White and black bars above the actograms indicate light (white) and dark (black) conditions. Arrowheads indicate the day when the firebrats were transferred from LD to DD. a and b indicated in the periodogram correspond to the analyzed time span, a and b, indicated in the actogram, respectively. An oblique line in the periodogram indicates a significance level of *P*<0.05; a peak value above the line was designated as significant. (A–D) Some of the *HR3* and *E75* RNAi firebrats showed a rhythm in LD, which disappeared upon transfer to DD. (E, F) Control firebrats injected with *DsRed2* dsRNA showed a significant rhythm throughout the recording period. No activity was recorded during the period indicated by double headed arrows because of molting. For further explanation, see text.

In order to examine the role of *Td'HR3* and *Td'E75* in the regulation of circadian behavioral rhythms, we compared locomotor rhythms between *Td'HR3* RNAi and *Td'E75* RNAi firebrats and control firebrats with *DsRed2* dsRNA injected in LD and DD (Fig. 3). Because *Td'HR3* RNAi and *Td'E75* RNAi firebrats showed a high lethality (Fig. 2E), we performed analysis of the locomotor activity rhythm only for firebrats survived for more than two weeks after the injection of dsRNA. The results are summarized in Table 3. Most of the *DsRed2* RNAi firebrats exhibited a nocturnal rhythm synchronizing to LD and free running in DD with a period of about 24 h (Fig. 3E and F), similar to intact animals [9], [10]. However, the free-running period considerably varied among individuals, showing rather large standard deviation (Table 3). In the *Td'HR3* RNAi and *Td'E75* RNAi firebrats, 100% (6/6) and 71% (12/17) of animals, respectively, exhibited, under LD, a rhythmic activity synchronized to the LD (Fig. 3A–D; Table 3), and the remaining animals were arrhythmic (Fig. 3B). In the ensuing DD, 75% (6/8) and 100% (4/4) animals for *Td'HR3* dsRNA#1 and #2 treatment, respectively, and 78% (7/9) and 83% (5/6) of animals for *Td'E75* dsRNA#1 and #2 treatment, respectively, almost immediately became arrhythmic (Fig. 3A–D; Table 3), suggesting that the rhythm observed in LD was a masking effect of the light. Interestingly, none of *Td'HR3* and *Td'E75* RNAi firebrats showed ecdysis. Thus, the interruption of the locomotor activity associated with ecdysis was not observed (Fig. 3A–D).

**Table 3:** Effects of ds*HR3*, ds*E75*, and ds*DsRed2* on the locomotor rhythm of the firebrat *Thermobia domestica*.

Treatment	n	No. of insects		Free-running period (mean ± SD) h
		Rhythmic (%)	Arrhythmic (%)	
LD				
* HR3* dsRNA#1	6	6 (100)	0 (0)	24.0±1.6
* E75* dsRNA#1	17	12 (71)	5 (29)	23.8±1.0
* DsRed2* dsRNA	20	18 (90)	2 (20)	23.9±0.3
DD				
* HR3* dsRNA#1	8	2 (25)	6 (75)^a^	24.2±0,6
* HR3* dsRNA #2	4	0 (0)	4(100)^b^	-
* E75* dsRNA #1	9	2 (22)	7 (78)^b^	24.5±0.4
* E75* dsRNA #2	6	1 (17)	5 (83)^b^	24.3
* DsRed2* dsRNA	24	18 (75)	6 (25)	24.1±0.8

a *P*<0.05, ^b^ *P*<0.001 vs *DsRed2* dsRNA, Chi-square test; SD, standard deviation; LD, light–dark cycle; DD, constant darkness.

### *Td'HR3* RNAi and *Td'E75* RNAi affect the expression of *Td'cyc*, *Td'tim*, and *Td'Clk*

**Figure 4:**
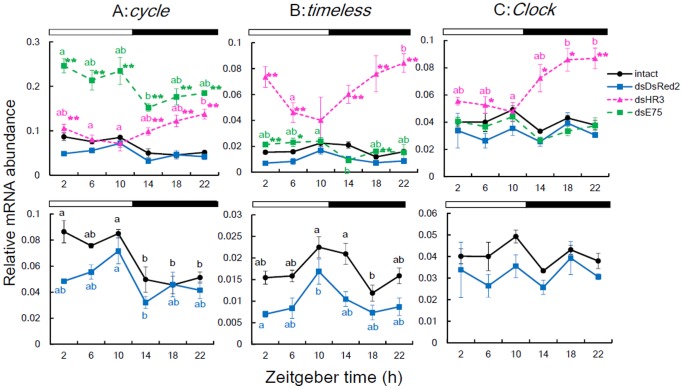
Effects of *HR3* (ds*HR3*, pink symbols) and *E75* dsRNA (ds*E75*, green symbols) on mRNA levels of *cyc*, *tim*, and *Clk* in firebrats. Values for intact and ds*DsRed2* injected control are shown in black and blue symbols, respectively, and replotted in lower panels with an expanded ordinate. (A) *cyc* normally peaked during the day. ds*E75* significantly upregulated the mRNA levels, while ds*HR3* shifted the rhythm to peak late at night. (B) *tim* showed a rhythm with a peak at late day or early night in intact firebrats. Ds*E75* slightly advanced the rhythm while ds*HR3* upregulated and shifted the rhythm to peak late at night. (C) *Clk* showed no significant rhythm in intact firebrats. Ds*E75* had no significant effect on *Clk* mRNA levels, while ds*HR3* induced a rhythm with a peak at mid–late night. *P<0.05, **P<0.01, *t* test vs ds*DsRed2* (exact *P* values are shown in S3 Table). Values with different letters significantly differ from each other (p<0.05, ANOVA with Tukey test, S4 Table). For *tim* in firebrats treated with ds*HR3* (B), significant rhythm was confirmed by the single cosinor method [28] and significant difference was found between values at ZT6 and ZT22 (*t*-test, P<0.05). For further explanation, see text and Fig. 2.

We next examined whether *Td'HR3* and/or *Td'E75* were regulators of *Td'cyc* transcription by measuring the daily expression profiles of *Td'cyc* mRNA in *Td'HR3* RNAi and *Td'E75* RNAi firebrats. For this and the following analyses only *Td'HR3* dsRNA#1 and *Td'E75* dsRNA#1 were used. *Td'E75* RNAi upregulated the *Td'cyc* mRNA level more than three-fold of the negative control but did not alter its rhythmic expression, while *Td'HR3* RNAi reversed the phase of the *Td'cyc* mRNA rhythm, with a gradual increase throughout the night to peak late at night (Fig. 4A, S3 and S4 Tables). These results demonstrate the involvement of *Td'HR3* and *Td'E75* in the circadian transcriptional regulation of *Td'cyc*.

*Td'HR3* and *Td'E75* RNAi considerably affected the circadian expression of the clock genes *Td'tim* and *Td'Clk*. *Td'HR3* RNAi upregulated the expression of *Td'tim* and shifted the rhythms to peak late at night, while *Td'E75* RNAi slightly increased the *Td'tim* mRNA levels to peak during the day (Fig. 4B, S3 and S4 Tables). Surprisingly, *Td'HR3* RNAi induced the rhythmic expression of *Td'Clk* to peak late at night with the upregulation of mRNA levels (Fig. 4C, S3 and S4 Tables), suggesting that the firebrat has a mechanism for the rhythmic expression of *Td'Clk*, which may be concealed by a mechanism involving *Td'HR3*. *Td'E75* RNAi, however, had no significant effect on *Td'Clk* mRNA levels (Fig. 4C, S3 Table). Interestingly, in *Td'HR3* RNAi firebrats, the rhythms of *Td'cyc*, *Td'tim*, and *Td'Clk* peaked almost in phase late at night, whereas in *Td'E75* RNAi firebrats, *Td'cyc* and *Td'tim* showed in-phase oscillations and peaked during the day (Fig. 4A–C).

### Rhythmic expressions of *Td'HR3* and *Td'E75* require *Td'cyc*, *Td'tim*, and *Td'Clk*

**Figure 5:**
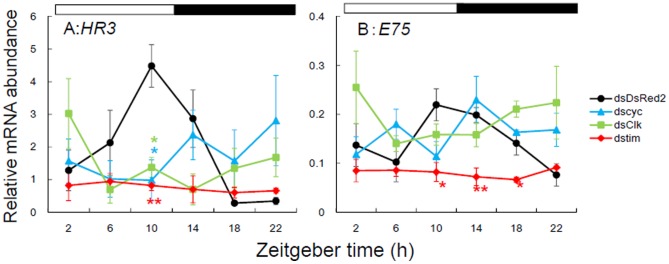
Effects of *cyc*, *Clk*, and *tim* dsRNA (ds*cyc*, ds*Clk*, and ds*tim*) on mRNA levels of *HR3* and *E75* in firebrats. (A) Ds*cyc*, ds*Clk*, and ds*tim* abolished the rhythmic expression of *HR3* with significant downregulation of mRNA levels. (B) RNAi of the three genes abolished the rhythm of *E75*. Ds*tim* effectively downregulated *E75* mRNA but ds*cyc* and ds*Clk* had no significant effects, except at ZT18 where a significant increase was observed. *P<0.05, **P<0.01, *t* test vs ds*DsRed2*. Data for ds*DsRed2* firebrats are replotted from Fig. 2. For further explanation, see text and Fig. 2.

BMAL1 and CLK have been postulated to be positive regulators of *Rorα* and *Rev-erbα* in mammalian clocks [15], [16]. This hypothesis prompted us to examine whether *Td'*CYC and *Td'*CLK activate *Td'HR3* and *Td'E75* transcriptions in the firebrat. Therefore, we measured their daily expression profiles in *Td'cyc* RNAi and *Td'Clk* RNAi firebrats. The mRNA levels of *Td'HR3* were significantly reduced and its circadian rhythm was abolished in both *Td'cyc* RNAi and *Td'Clk* RNAi firebrats (Fig. 5A). However, for *Td'E75*, its rhythm was disrupted in both ds*Clk* and ds*cyc* treatments but no significant changes were observed in its mRNA levels (Fig. 5B).

We also performed the same measurement in *Td'tim* RNAi firebrats because we speculated that the PER/TIM heterodimer, similar to the PER/CRY complex in mammals, may suppress *Td'HR3* and *Td'E75* transcription indirectly by repressing the transcriptional activity of CYC/CLK. The levels of both *Td'HR3* and *Td'E75* mRNAs were significantly downregulated and their rhythms were disrupted in *Td'tim* RNAi firebrats (Fig. 5A, B). This result is inconsistent with the mammalian clock hypothesis but is explained by our earlier finding that *Td'tim* RNAi downregulates *Td'cyc* transcript levels [10].

### 20E upregulates *Td'HR3* and *Td'E75* transcription

**Figure 6:**
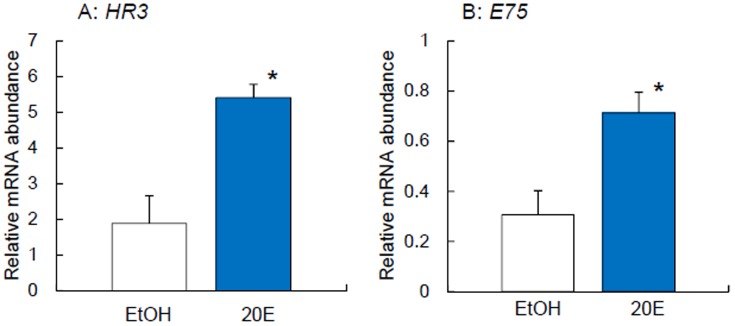
Effects of ecdysone on mRNA levels of *Td'HR3* (A) and *Td'E75* (B) in firebrats. White and blue columns indicate the mRNA levels of EtOH (negative control) and ecdysone injected firebrats, respectively. 100% EtOH (69 nl) or 34.5 ng ecdysone (dissolved in 69 nl of EtOH) was injected and firebrats were collected at ZT 10 (10 h after light on) one week after the injection. The abundance of mRNA was measured by qPCR. The data collected from 4 independent experiments were averaged and plotted as mean ± SEM values relative to the value of *rp49* mRNA used as reference.*P<0.05, *t*-test.

Given that *HR3* and *E75* genes expression profiles in *D. melanogaster* strongly correlated with ecdysis [20], we examined the effects of 20E on *Td'HR3* and *Td'E75* mRNA levels. We injected 34.5 ng of 20E into the abdomen of adult firebrats. *Td'HR3* and *Td'E75* were evidently upregulated 1 week after 20E injection compared with the control firebrats injected with EtOH (Fig. 6).

## Discussion

The present study revealed that the mRNAs of the nuclear receptor genes *Td'HR3* and *Td'E75* were rhythmically expressed in both LD and DD, peaking late day to early night (Fig. 2). The rhythmic expression was comparable to their mammalian orthologs, *Rorα* and *Rev-erbα* [15], [16]. Since RNAi of *Td'cyc* and *Td'Clk* disrupted the daily expression rhythms of *Td'HR3* and *Td'E75* genes, and downregulated *Td'HR3* mRNA levels (Fig. 5), we assume that the rhythmic expression of *Td'HR3* and *Td'E75* are regulated by *Td'*CYC and *Td'*CLK, similar to their mammalian orthologs.

Our results demonstrated that *Td'HR3* and *Td'E75* are factors involved in the cyclic transcription of *Td'cyc* in the firebrat. The RNAi experiment showed that *Td'*E75 may be a repressor of *Td'cyc* transcription, similar to mammalian REV-ERBα [15], because its RNAi resulted in substantial upregulation of *Td'cyc* mRNA levels (Fig. 4A). However, *Td'*HR3 may act as a phase regulator of *Td'cyc* since its RNAi resulted in a phase reversal in *Td'cyc* oscillation with an increase of mRNA levels during the night (Fig. 4A). The results also imply that this may occur through another unknown factor(s) that activates the transcription of *Td'cyc* during the night.

In addition, *Td'HR3* might also participate in the regulation of *Td'Clk* expression, since its RNAi induced a circadian oscillation and an upregulation of *Td'Clk* mRNA levels (Fig. 4C). Interestingly, *Td'cyc*, *Td'Clk*, and *Td'tim* oscillated in phase to peak around mid–late night in the *Td'HR3* RNAi firebrats (Fig. 4A–C). However, in the *Td'E7*5 RNAi firebrats, *Td'cyc* and *Td'tim* showed an in-phase oscillation that peaked during the day (Fig. 4A–B), suggesting that *Td'E75* regulates the phase of *Td'tim*. The phase coherence was never observed in intact firebrats; *Td'cyc* and *Td'tim* oscillated with differential peaks phased during the day and late day to early night, respectively. Their in-phase oscillation in RNAi firebrats suggests that their transcriptions are regulated by a common factor, and *Td'*HR3 and *Td'*E75 play a role as phase regulators to directly or indirectly set the clock gene oscillations in a proper phase relationship. A similar phase coherence was observed in the sandfly *Lutzomyia longipalpis*, in which *Clk* and *cyc* transcripts peaked simultaneously with or slightly earlier than *per* and *tim* [21], [22], and in some mammalian peripheral tissues where *Bmal1* and *Per* were expressed almost in phase [23], [24]. This feature is in opposition to the hypotheses of the *Drosophila* and mammalian central clocks, where peaks of *per/tim* (*Per/Cry*) and *Clk/cyc* (*Clk/Bmal1*) transcriptions are about 12 h apart from each other [25]. The reason and mechanism underlying the phase coherence should be addressed in future studies.

The behavioral analysis of *Td'HR3* RNAi and *Td'E75* RNAi firebrats revealed that both genes have two roles. First, they are important for the persistence of locomotor activity rhythms in DD (Fig. 3A–B; Table 3), which is also seen in mice where RORα and REV-ERBα are required for normal daily locomotor activity [15], [16], [26]. The *Td'HR3* RNAi or *Td'E75* RNAi firebrats showed in-phase oscillations of *Td'cyc, Td'tim*, and *Td'Clk* or *Td'cyc* and *Td'tim*, respectively (Fig. 4A–C). Nevertheless, they lost the locomotor rhythm in DD (Fig. 3A–B). This result suggests that the persistence of the locomotor rhythm requires a temporally coordinated and/or properly leveled expression of those clock genes in the firebrat. Thus, *HR3* and *E75* might have been involved as essential components in the ancestral insect clock. In addition, they are known to play important roles within the ecdysone-signaling pathway in *Drosophila* [20], and this may be true in the firebrat because they were upregulated by 20E (Fig. 6). This hypothesis also explains why a majority of firebrats with *Td'HR3* and *Td'E75* RNAi died from ecdysis failure (Fig. 2E), although no such failure was observed in the RNAi of other clock- and clock-related genes [9], [10]. Thus, they may couple the developmental processes and circadian rhythms in the firebrat.

The circadian clock machinery has been substantially clarified in *Drosophila* and mammals. In the current *Drosophila* clock model, *per* and *tim* are rhythmically expressed by the core-feedback loop consisting of transcriptional repressors, PER and TIM, and activators, CYC and CLK [27]. *Clk* is also rhythmically expressed by a loop, including *vri* and *Pdp1ε* [6], [8]. It seems likely that the firebrat's core loop might be operating in a similar way. *Td'*TIM may have two roles, i.e., repression of the transcriptional activity of *Td'*CYC and activation of the *Td'cyc* transcription through some unknown mechanism, because our previous results have shown that *Td'tim* RNAi downregulates the *Td'cyc* transcripts [10]. This scheme also explains why *Td'tim* RNAi downregulated both *Td'HR3* and *Td'E75* (Fig. 5). In contrast to *Drosophila*, *Td'cyc* is rhythmically expressed in the firebrat [9], [10], and our results revealed that the oscillatory mechanism involved *Td'HR3* and *Td'E75* although the details have yet to be clarified. This property resembles the mammalian clock.

Unexpectedly, *Td'HR3* RNAi revealed the rhythmic expression of *Td'Clk*, suggesting the coexistence of an oscillatory mechanism for *Td'Clk*. In comparison to the *Drosophila* circadian clock, we currently hypothesize that the rhythmic transcription of *Td'Clk* is regulated by VRI and PDP1ε. In many insects, *cyc* but not *Clk*, has been shown to be rhythmically expressed. However, our data suggest that, even in those species, the mechanism for the cyclic expression of *Clk* might be retained but concealed in normal physiological conditions. In fact, we have recently shown that *Clk* is rhythmically expressed when *cyc* is knocked down in the cricket *G. bimaculatus* [12]. Interestingly, both *Clk* and *cyc* are expressed in a rhythmic manner in the sandflies [21], [22]. Together with these facts, our results suggest that an ancestral insect clock has mechanisms for the rhythmic expression of both *cyc* and *Clk* but have diversified to express either or both of them during the course of adaptation to specific environments. Further comparative studies are necessary to test this view.

## Supporting Information

S1 Table **Results of one way ANOVA for daily changes of mRNA levels of** ***HR3*** **and** ***E75*** **in intact firebrats and those treated with dsRNAs of** ***DsRed2*** **,** ***HR3*** **and** ***E75*** **.**
(PDF)

S2 Table **P values of** ***t*** **-test between** ***HR3*** **or** ***E75*** **mRNA levels of firebrats treated with ds** ***HR3*** **or ds** ***E75*** **and those treated with ds** ***DsRed2*** **.**
(PDF)

S3 Table **P values of t-test between firebrats treated with ds** ***HR3*** **or ds** ***E75*** **and those treated with ds** ***DsRed2*** **.**
(PDF)

S4 Table **Results of one way ANOVA for daily changes in mRNA levels of** ***timeless*** **,** ***cycle*** **and** ***Clock*** **genes in intact firebrats and those treated with dsRNAs of** ***DsRed2*** **,** ***HR3*** **and** ***E75*** **.**
(PDF)
