# Supplementary material for: The Nuclear Receptor Genes HR3 and E75 Are Required for the Circadian Rhythm in a Primitive Insect
Source: PLoS One. 2014 Dec 11;9(12):e114899. doi: 10.1371/journal.pone.0114899 (PMC4263706; doi:10.1371/journal.pone.0114899)
Supplement: S1 Table — Results of one way ANOVA for daily changes of mRNA levels of HR3 and E75 in intact firebrats and those treated with dsRNAs of DsRed2 , HR3 and E75 . (PDF) [file pone.0114899.s001.pdf]

Table S1. Results of one way ANOVA for daily changes of mRNA levels of *HR3* and *E75* in intact firebrats and those treated with dsRNAs of *DsRed2*, *HR3* and *E75*.

Gene	Treatment	*F*	*P*
HR3	intact LD	$F_{5,12}$ = 6.7042	0.003353
	intact DD	$F_{5,14}$ = 3.0036	0.04774
	ds*HR3*#1	$F_{5,12}$ = 0.5105	0.7634
	ds*HR3*#2	$F_{2,18}$ = 0.1876	0.8306
	ds*DsRed2*	$F_{5,12}$ = 6.0474	0.005078
E75	intact LD	$F_{5,12}$ = 4.1136	0.0208
	intact DD	$F_{5,14}$ = 4.7909	0.007238
	ds*E75*#1	$F_{5,12}$ = 1.8049	0.1863
	ds*E75*#2	$F_{2,8}$ = 1.8489	0.2187
	ds*DsRed2*	$F_{5,14}$ = 3.1068	0.043
