# Supplementary material for: The Nuclear Receptor Genes HR3 and E75 Are Required for the Circadian Rhythm in a Primitive Insect
Source: PLoS One. 2014 Dec 11;9(12):e114899. doi: 10.1371/journal.pone.0114899 (PMC4263706; doi:10.1371/journal.pone.0114899)
Supplement: S2 Table — P values of t -test between HR3 or E75 mRNA levels of firebrats treated with ds HR3 or ds E75 and those treated with ds DsRed2 . (PDF) [file pone.0114899.s002.pdf]

Table S2. *P* values of *t*-test between *HR3* or *E75* mRNA levels of firebrats treated with ds*HR3* or ds*E75* and those treated with ds*DsRed2*.

Gene	Treatment	ZT2	ZT6	ZT10	ZT14	ZT18	ZT22
HR3	ds*HR3*#1	0.295019	0.87427	0.008929	0.590062	0.07529	0.069051
	ds*HR3*#2	-	0.093625	0.030855	0.834946	-	-
E75	ds*E75*#1	0.750355	0.995857	0.043406	0.002956	0.208633	0.333817
	ds*E75*#2	-	0.236622	0.001241	0.005256	-	-
