# Supplementary material for: The Nuclear Receptor Genes HR3 and E75 Are Required for the Circadian Rhythm in a Primitive Insect
Source: PLoS One. 2014 Dec 11;9(12):e114899. doi: 10.1371/journal.pone.0114899 (PMC4263706; doi:10.1371/journal.pone.0114899)
Supplement: S3 Table — P values of t-test between firebrats treated with ds HR3 or ds E75 and those treated with ds DsRed2 . (PDF) [file pone.0114899.s003.pdf]

Table S3. *P* values of *t*-test between firebrats treated with ds*HR3* or ds*E75* and those treated with ds*DsRed2*.

Gene	*treatment*	ZT2	ZT6	ZT10	ZT14	ZT18	ZT22
cycle	ds*HR3*	0.0031	0.0974	0.9844	0.0032	0.0033	0.0004
	ds*E75*	0.0002	0.0023	0.0021	0.0003	0.0008	5.675e-06
timeless	ds*HR3*	0.0012	0.0086	0.2627	0.0022	0.0081	0.0005
	ds*E75*	0.0005	0.0204	0.1605	0.5336	0.0089	0.3589
Clock	ds*HR3*	0.1732	0.0328	0.2289	0.0115	0.0148	0.0018
	ds*E75*	0.6325	0.1867	0.2571	0.8135	0.5256	0.3143
