# Supplementary material for: The Nuclear Receptor Genes HR3 and E75 Are Required for the Circadian Rhythm in a Primitive Insect
Source: PLoS One. 2014 Dec 11;9(12):e114899. doi: 10.1371/journal.pone.0114899 (PMC4263706; doi:10.1371/journal.pone.0114899)
Supplement: S4 Table — Results of one way ANOVA for daily changes in mRNA levels of timeless , cycle and Clock genes in intact firebrats and those treated with dsRNAs of DsRed2 , HR3 and E75 . (PDF) [file pone.0114899.s004.pdf]

Table S4. Results of one way ANOVA for daily changes in mRNA levels of *timeless, cycle* and *Clock* genes in intact firebrats and those treated with dsRNAs of *DsRed2, HR3 and E75* .

Gene	Treatment	*F*	*P*
timeless	intact	$F_{5,16}$ = 4.0789	0.01406
	ds*DsRed2*	$F_{5,12}$ = 3.3111	0.04143
	ds*HR3*	$F_{5,12}$ = 2.551	0.08534
	ds*E75*	$F_{5,12}$ = 3.3256	0.04089
cycle	intact	$F_{5,12}$ = 8.6729	0.001117
	ds*DsRed2*	$F_{5,15}$ = 3.2328	0.03534
	ds*HR3*	$F_{5,12}$ = 4.538	0.01484
	ds*E75*	$F_{5,12}$ = 3.7757	0.02756
Clock	intact	$F_{5,17}$ = 2.641	0.06071
	ds*DsRed2*	$F_{5,12}$ = 0.5853	0.7114
	ds*HR3*	$F_{5,12}$ = 5.5997	0.006852
	ds*E75*	$F_{5,12}$ = 2.4437	0.09507
